# Precision nutrition management in hyperlipidemia-associated acute pancreatitis: mechanistic insights and personalized therapeutic approaches

**DOI:** 10.3389/fnut.2025.1583889

**Published:** 2025-06-18

**Authors:** Jingyuan Ma, Xing Wan, Jifeng Liu, Xuyang Hu, Yanna Ma, Yunhai Gao

**Affiliations:** ^1^The First Clinical Medical College, Liaoning University of Traditional Chinese Medicine, Shenyang, China; ^2^The First Affiliated Hospital of Dalian Medical University, Dalian, China; ^3^Institute of Integrated Traditional Chinese and Western Medicine, Dalian Medical University, Dalian, China; ^4^The Second Clinical Medical College, Liaoning University of Traditional Chinese Medicine, Shenyang, China

**Keywords:** hyperlipidemia, acute pancreatitis, precision nutrition, clinical nutrition, mechanism, multi-omics integration, personalized medicine

## Abstract

Hyperlipidemia-associated acute pancreatitis (HLAP), an acute inflammatory disorder triggered by dyslipidemia, has witnessed a rising global incidence with significant health implications. The pathogenesis of HLAP involves complex interactions among lipid metabolism dysregulation, inflammatory cascades, and oxidative stress. Conventional therapeutic approaches, while providing partial symptomatic relief, exhibit limitations in addressing individual variability. Precision nutrition management emerges as a novel paradigm integrating multi-omics profiling (genomic, metabolomic) and clinical parameters to develop personalized intervention strategies. This comprehensive review analyzes the pathophysiological mechanisms linking lipid dyshomeostasis to HLAP progression, systematically evaluates the scientific foundation for precision nutrition interventions, and identifies key gaps in current implementation strategies. Furthermore, we examine current research limitations and outline future avenues for enhancing therapeutic efficacy via personalized nutritional interventions.

## 1 Introduction

Acute pancreatitis (AP), a gastrointestinal emergency with multifactorial etiology, demonstrates increasing epidemiological association with hyperlipidemia (1). Globally, hyperlipidemia accounts for 10% of AP cases, characterized by heightened clinical severity and elevated recurrence rates (2). In China, hyperlipidemia has surpassed alcohol consumption to become the second most prevalent AP etiology following cholelithiasis (3). A retrospective Chinese cohort study (2001–2016, *n* = 475 moderate–severe AP patients) revealed 108 HLAP cases (22.7%), with HLAP prevalence increasing from 14.3 to 35.5% during the study period, contrasting with declining rates of biliary pancreatitis (4). Epidemiological trends further show a 2.6-fold increase in HLAP incidence over the past decade, coinciding with the global rise in metabolic syndrome and obesity (5).

The pathognomonic feature of HLAP involves serum triglyceride (TG) concentrations exceeding 11.30 mmol/L (1,000 mg/dL) after excluding biliary, alcoholic, and other etiologies (6). Mechanistically, excessive TG hydrolysis generates cytotoxic free fatty acids that induce pancreatic capillary endothelial damage and acinar cell apoptosis (7). Clinically, HLAP demonstrates greater propensity for progression to necrotizing pancreatitis compared to other AP subtypes (8). Population-level analyses reveal a dose-dependent relationship between hypertriglyceridemia and AP risk: each 100 mg/dL increment above normal TG levels (150 mg/dL) elevates AP risk by 4%, with exponential risk escalation beyond 500 mg/dL (9).

The hypercatabolic state in early HLAP induces rapid-onset negative nitrogen balance and hypoalbuminemia, exacerbating malnutrition while compounding gastrointestinal complications including abdominal pain, intestinal mucosal barrier dysfunction, and malabsorption (10). Traditional nutritional interventions, although temporarily alleviating hyperlipidemia and inflammation, lack personalization and frequently result in suboptimal, inconsistent clinical outcomes. This is primarily due to the inadequate integration of individual metabolic phenotypes, genetic susceptibilities, and inflammatory profiles (11).

The advent of precision medicine has catalyzed paradigm shifts in therapeutic approaches. Precision nutrition management employs multi-omics integration (genomic, metabolomic, and clinical data) to formulate tailored dietary regimens (12). This strategy shows particular promise in HLAP management by addressing individual variations in lipid metabolism pathways, inflammatory responses, and nutrient utilization efficiency. Compared to conventional one-size-fits-all approaches, precision nutrition offers mechanistic-driven solutions to optimize therapeutic outcomes and prevent disease recurrence.

## 2 Pathogenesis of HLAP

**Figure 1:**
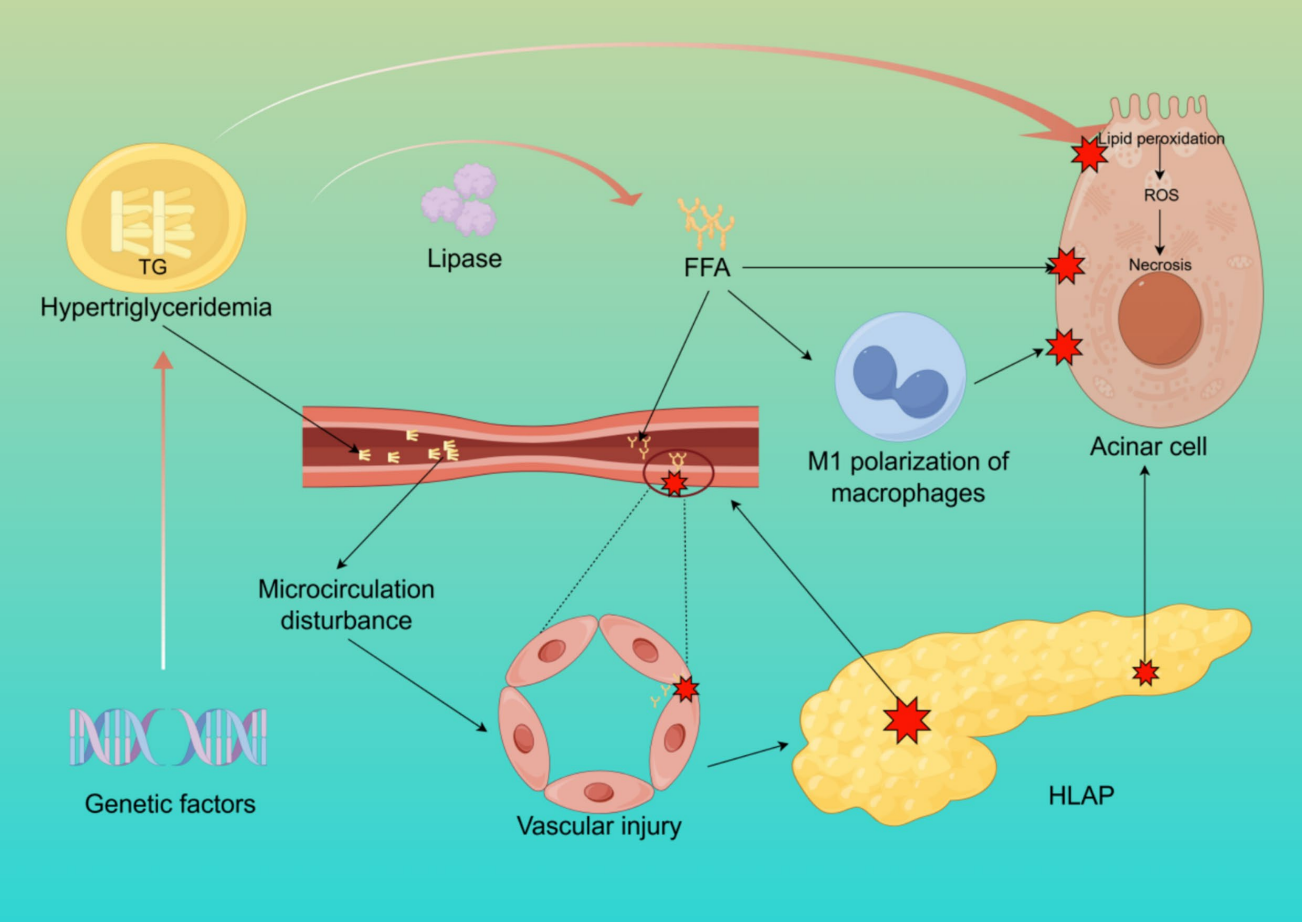
The pathogenesis of HLAP. This figure illustrates the core mechanisms of HLAP, including elevated triglyceride hydrolysis into free fatty acids, acinar cell injury, endothelial dysfunction, oxidative stress, and activation of inflammatory pathways. Created with Figdraw.com.

The pathophysiological mechanisms of HLAP are complex, involving dysregulated lipid metabolism, inflammation activation, pancreatic microcirculatory disturbances, oxidative stress, and insulin resistance, among multiple interacting pathways (13–16). The core pathological processes revolve around excessive free fatty acids (FFA) release, microcirculatory damage, and the amplification of systemic inflammatory responses (Figure 1).

### 2.1 Cytotoxicity of FFA

In hyperlipidemic conditions, elevated TG levels in the bloodstream are hydrolyzed by pancreatic lipase, releasing large amounts of FFA. These FFA, primarily composed of palmitic acid (PA), are unsaturated fatty acids (17). Under normal conditions, FFA bind to plasma albumin for systemic transport and oxidation. However, when the concentration of FFA exceeds the binding capacity of albumin, excess FFA remain unbound in the bloodstream, leading to cytotoxic effects on pancreatic acinar and vascular endothelial cells through lipid peroxidation of cell membranes (18). Studies have shown that unsaturated fatty acids exhibit higher toxicity than saturated fatty acids (19). Damage to pancreatic capillaries results in pancreatic ischemia, intracellular pH reduction in acinar cells, and the formation of an acidic environment, which further activates trypsinogen and enhances FFA toxicity, leading to autodigestion of pancreatic acinar cells. Additionally, excessive FFA and TG levels increase blood viscosity, further impairing pancreatic circulation and exacerbating local inflammation (20). Research on isolated pancreatic acinar cells has demonstrated that exposure to FFA significantly elevates levels of hydroperoxidized phosphatidylcholine, indicating that FFA mediate lipid peroxidation and disrupt cell membranes, causing direct injury to pancreatic acinar cells (21). Another study observed that adding FFA to cultured rat pancreatic acinar cells resulted in damage that was positively correlated with both FFA concentration and exposure duration (22). Excessive FFA also induce M1 polarization of macrophages and mediate inflammatory responses and pyroptosis in pancreatic acinar cells (23).

### 2.2 Pancreatic microcirculatory disturbances

Microcirculation refers to the network responsible for the transport of substances, energy, and signals at the tissue and cellular levels. Proper microcirculatory perfusion is crucial for maintaining normal physiological metabolism in organs (24). Hyperlipidemia increases plasma viscosity and reduces erythrocyte deformability, leading to decreased pancreatic microcirculatory perfusion (25). Furthermore, excessive FFA accumulation promotes platelet activation and the coagulation cascade, resulting in microthrombus formation, which aggravates pancreatic ischemia and hypoxia (8). Ischemic injury can cause acinar cell necrosis, exacerbating local inflammation and edema, and in severe cases, leading to necrotizing pancreatitis (26). Pancreatic microcirculatory disturbances manifest as chylomicron aggregation blocking capillaries, leading to abnormal hemorheology, hypercoagulation, and microthrombosis, as well as increased vascular permeability due to microvascular spasms and endothelial damage. Additionally, ischemia–reperfusion injury contributes to oxidative stress through increased free radical generation (27, 28).

### 2.3 Oxidative stress and inflammatory cascade response

Oxidative stress refers to an imbalance between oxidative and antioxidative processes within the body, with lipid peroxidation playing a pivotal role in HLAP progression (29). Excessive triglyceride oxidation in the pancreas generates reactive oxygen species (ROS) and lipid peroxidation products, further damaging DNA, proteins, and lipids, thereby exacerbating pancreatic injury (30). Moreover, FFA stimulate macrophage activation, triggering a systemic inflammatory response that leads to a “cytokine storm,” which can ultimately result in multiple organ dysfunction syndrome (MODS) and increased HLAP mortality (31). Animal studies have demonstrated that alleviating oxidative stress-related pancreatic damage can mitigate AP severity (32). Oxidative stress activation promotes the recruitment of inflammatory cells, further aggravating pancreatic tissue damage, whereas antioxidant therapy significantly reduces pancreatic and other organ injuries associated with AP (33).

### 2.4 Genetic and acquired lipid metabolism defects

Familial hypertriglyceridemia (HTG) has long been recognized as a disorder characterized by increased very-low-density lipoprotein (VLDL) particles and an autosomal dominant inheritance pattern (34). In the genomic era, it has been established that while familial HTG can be clustered within families, it is a polygenic disorder, with phenotypic expression influenced by environmental factors. Clinically, it is characterized by moderate HTG, which increases cardiovascular risk, while in rare cases, it presents with severe HTG and an elevated risk of AP (35). Some HLAP patients exhibit lipid metabolism-related genetic mutations, such as mutations in the LPL and APOC2 genes, which lead to impaired chylomicron clearance, resulting in familial chylomicronemia and significantly increasing the risk of HLAP (36–39). Understanding these genetic factors is crucial for identifying high-risk individuals and implementing targeted preventive strategies.

## 3 Theoretical basis of precision nutrition management

An increasing number of studies suggest that an individual’s genetic background significantly influences their response to dietary interventions (40). For instance, the ability to digest lactose in adulthood is more common in Northern Europeans than in East Asians (41). Therefore, precise nutritional interventions should be tailored based on an individual’s genotype, metabolic phenotype, and lifestyle to enhance intervention effectiveness and prevent and treat related diseases (42).

### 3.1 Association between genomics and nutritional interventions

Recent genomic studies indicate that genes related to lipid metabolism, inflammatory responses, and autophagy regulation in pancreatic cells play crucial roles in the development and progression of HLAP (29, 43). Specific gene variants, such as APOE and LPL mutations, have been shown to be closely associated with hyperlipidemia and pancreatitis (44–46). These genes are critical in lipid metabolism, cholesterol transport, and pancreatic cell injury repair. Polymorphisms in the FTO gene have been linked to fat accumulation and obesity, while variations in the APOA5 gene affect triglyceride metabolism and regulate lipid levels (47, 48). Nutrigenomics explores how dietary factors influence gene expression, subsequently affecting protein and metabolite levels (49). The interaction between genes and diet may play a vital role in metabolic and inflammatory responses, providing a theoretical foundation for developing precision nutritional intervention strategies.

### 3.2 Metabolomics as a new target for personalized interventions

Metabolomics is a high-throughput analytical science that systematically identifies and quantifies small-molecule metabolites (<1,500 Da) within biological systems to understand their dynamic changes in response to genetic, environmental, or pathological perturbations. It plays a key role in precision nutrition development, primarily focusing on food intake biomarkers, metabolic phenotypes, and responses to interventions (50). Studies have revealed metabolic reprogramming in multiple pathways in HLAP patients, particularly in fatty acid metabolism, cholesterol metabolism, and amino acid metabolism (14, 51, 52). For instance, abnormal elevations in triglycerides (TG) and low-density lipoprotein (LDL), along with imbalances in insulin resistance markers (e.g., branched-chain amino acids) and oxidative stress products (e.g., malondialdehyde), provide potential targets for personalized interventions (53, 54). These metabolic features not only reveal core disease mechanisms—such as chylomicronemia-induced pancreatic microcirculatory disturbances—but also guide targeted nutritional regulation strategies, such as increasing omega-3 fatty acids to modulate lipid metabolism, supplementing dietary fiber to enhance short-chain fatty acid (SCFA) production by gut microbiota, or using antioxidant nutrients (e.g., vitamin C) to mitigate oxidative damage (55–58). Comprehensive metabolite analysis in patients enables a more accurate assessment of individual metabolic states, providing a scientific basis for personalized nutritional interventions.

### 3.3 Integration of microbiome and precision nutrition management

Emerging research suggests that the microbiome can potentially influence human physiology by participating in digestion, nutrient absorption, mucosal immune responses, and the synthesis or regulation of various bioactive compounds (59–61). Consequently, diet-induced microbial changes may contribute to disease onset and progression (62). The gut microbiota plays a significant role in the development of AP, characterized by a reduction in beneficial bacteria and an increase in opportunistic pathogens. This imbalance leads to decreased SCFA secretion and epithelial damage, thereby compromising the intestinal mucosal barrier (63). One study found that 59% of AP patients exhibited intestinal barrier damage and increased mucosal permeability, leading to bacterial translocation, pancreatic necrosis, infection, and multiple organ dysfunction syndrome (MODS) (64). A randomized controlled trial demonstrated that probiotics improved gut barrier function and modulated microbiota composition in severe AP (SAP) patients, reducing inflammatory cytokine levels, alleviating abdominal pain, mitigating pancreatic edema, and shortening bowel movement recovery time and hospital stays (65). Similar results were observed in another randomized controlled trial involving mild AP (66). Soluble dietary fiber (SDF) influences intestinal integrity and regulates gut microbiota (67). A single-blind randomized controlled study found that SDF reduced the time needed to achieve energy targets during enteral nutrition (EN), improved gut permeability and motility disorders, and decreased feeding intolerance in SAP patients (68). Therefore, the microbiome provides a “microbiota-metabolism” regulatory target for the personalized nutritional design of HLAP, advancing a more precise disease management model.

## 4 Precision nutritional interventions for HLAP

### 4.1 Conventional nutritional support

The primary management goals in the acute phase of HLAP are to minimize pancreatic stimulation, control inflammatory responses, and support energy metabolism. Previous studies have suggested that higher serum triglyceride (TG) levels are associated with a greater tendency for HLAP to become severe, a shorter time to systemic inflammatory response syndrome (SIRS), and a higher incidence of multiple organ dysfunction syndrome (MODS) (69). During fasting in AP patients, negative nitrogen balance is common, making nutritional support an essential component of HLAP management (70). During the course of pancreatitis, pancreatic exocrine function is suppressed; thus, food intake or artificial nutrition does not stimulate exocrine secretion (71). For patients who can tolerate oral feeding, an initial low-fat solid diet is recommended (72). Early oral feeding may shorten hospital stays in these patients (73).

For patients with severe acute pancreatitis, appropriate clinical nutrition strategies are necessary, with enteral nutrition (EN) being preferred (74, 75). EN helps maintain intestinal mucosal integrity, stimulates intestinal motility, increases visceral blood flow, prevents bacterial overgrowth, and reduces microbial translocation (76). Studies have shown that initiating nutrition within 24–72 h of admission reduces bacterial translocation, thereby mitigating systemic inflammation while preserving intestinal integrity and microbiome composition (77). Compared to parenteral nutrition (PN), EN is more effective in maintaining gut barrier function and reducing the risk of infections and pancreatic complications (78). A systematic review provided strong evidence supporting the advantages of EN in reducing infectious complications and mortality in AP patients (79). Special formulations with low fat and low osmolarity are recommended to minimize the burden on pancreatic enzyme secretion. For patients without respiratory failure, who are conscious, free from nausea and vomiting, and without significant gastrointestinal obstruction, an oral nutrition trial should be initiated immediately (80). In summary, guidelines recommend that AP patients receive EN rather than PN unless contraindications or intolerance to EN exist (81, 82). The European Society for Clinical Nutrition and Metabolism (ESPEN) recommends early EN via nasogastric tube, with nasojejunal feeding preferred if intolerance occurs (82). Given that “waking up the gut” is more beneficial than “gut rest,” patients with mild HLAP should be allowed oral feeding within 24 h if tolerated. However, patients with moderate to severe HLAP (acute physiology and chronic health evaluation II (APACHE-I) >8) who experience hemodynamic instability and require vasopressor support are often unable to tolerate oral feeding due to the increased risk of non-occlusive mesenteric ischemia (3). For these patients, EN should be initiated via a feeding tube within 24 h of hemodynamic stabilization (83, 84). Studies have shown that the improvement in nutritional status and tolerance of EN in HLAP patients is related to the choice between nasogastric and nasojejunal feeding tubes (85). Currently, high-quality evidence comparing nasogastric and nasojejunal tube feeding is lacking. Although nasojejunal feeding reduces the risk of aspiration and pancreatic stimulation, its placement requires endoscopic and/or fluoroscopic guidance or specialized equipment. In contrast, nasogastric tubes are easier to insert and can be placed at the bedside (Table 1) (86).

**Table 1:** Comparison of conventional nutritional support strategies.

Intervention	Key points	Evidence basis	Clinical recommendations
Enteral nutrition (EN)	Maintains gut barrier function, reduces infection risk; low-fat, low-osmolarity formulas recommended	Reduces pancreatic complications and mortality (74–79)	Prioritize EN over PN (unless contraindicated); initiate within 24–72 h in acute phase
Parenteral nutrition (PN)	Reserved for EN intolerance/contraindications; may increase infection risk	Evidence supports EN over PN in reducing infections (78, 79)	Avoid early use; monitor metabolic complications
Early oral feeding	Shortens hospital stay; low-fat solid diet (mild HLAP) recommended	Reduces SIRS duration and MODS incidence (72, 73)	Initiate oral feeding within 24 h if hemodynamically stable without vomiting/obstruction
Nasogastric vs. nasojejunal tubes	Nasogastric tubes are easier to place; nasojejunal tubes reduce aspiration risk; no significant efficacy difference	Limited high-quality comparative evidence (85, 86)	Nasogastric as first-line; switch to nasojejunal if intolerance occurs

### 4.2 Immunonutrition support

Immunonutrition involves the addition of immune-enhancing nutrients to conventional nutrition, aiming to improve inflammatory responses, malnutrition, metabolic abnormalities, and immune imbalances (87, 88). Recent studies have highlighted the benefits of immunonutrition (89–91). Key immunonutrients include omega-3 fatty acids, glutamine, arginine, and nucleotides (92). Omega-3 fatty acids competitively inhibit the arachidonic acid metabolic pathway, reducing the synthesis of pro-inflammatory prostaglandins and leukotrienes, thereby mitigating pancreatic inflammation (93, 94). Glutamine, a non-essential amino acid, plays a role in protein synthesis, energy supply, and immune support, with increased demand during stress conditions (95). As the primary energy source for intestinal immune cells, glutamine helps maintain gut barrier integrity and reduces bacterial translocation and infections (96). However, studies comparing EN with and without glutamine supplementation have shown no significant advantage with glutamine (97, 98). Nonetheless, compared to standard PN, PN supplemented with glutamine and omega-3 fatty acids has been associated with improved outcomes (99, 100). Arginine, a conditionally essential amino acid found in meat, fish, and nuts, promotes nitric oxide (NO) synthesis, improves microcirculation, and enhances T-cell and macrophage function (101, 102). Some studies have linked immunonutrition to reduced AP mortality, lower infection rates, and shorter hospital stays (103). However, a meta-analysis found no significant benefit of immunonutrition over standard EN in terms of overall infection rates and mortality (104). The safety and efficacy of immunonutrition remain inconclusive, requiring further research (Table 2) (105).

**Table 2:** Immunonutrients: mechanisms and evidence status.

Immunonutrient	Mechanism	Research Findings	Current Evidence Status
Omega-3 fatty acids	Inhibit pro-inflammatory prostaglandins; modulate lipid metabolism	Reduce inflammatory markers; improve outcomes in severe cases (93, 94, 99, 100)	Effective in PN; inconclusive for EN
Glutamine	Primary energy source for gut immune cells; maintains barrier integrity	Reduces infections in PN; no significant benefit in EN (95–100)	Recommended for PN; insufficient evidence for EN
Arginine	Promotes NO synthesis; enhances T-cell function	Mixed results: some studies show reduced mortality, meta-analyses show no benefit (101–104)	Safety concerns; requires individualized use

### 4.3 Precision nutrition strategies

**Figure 2:**
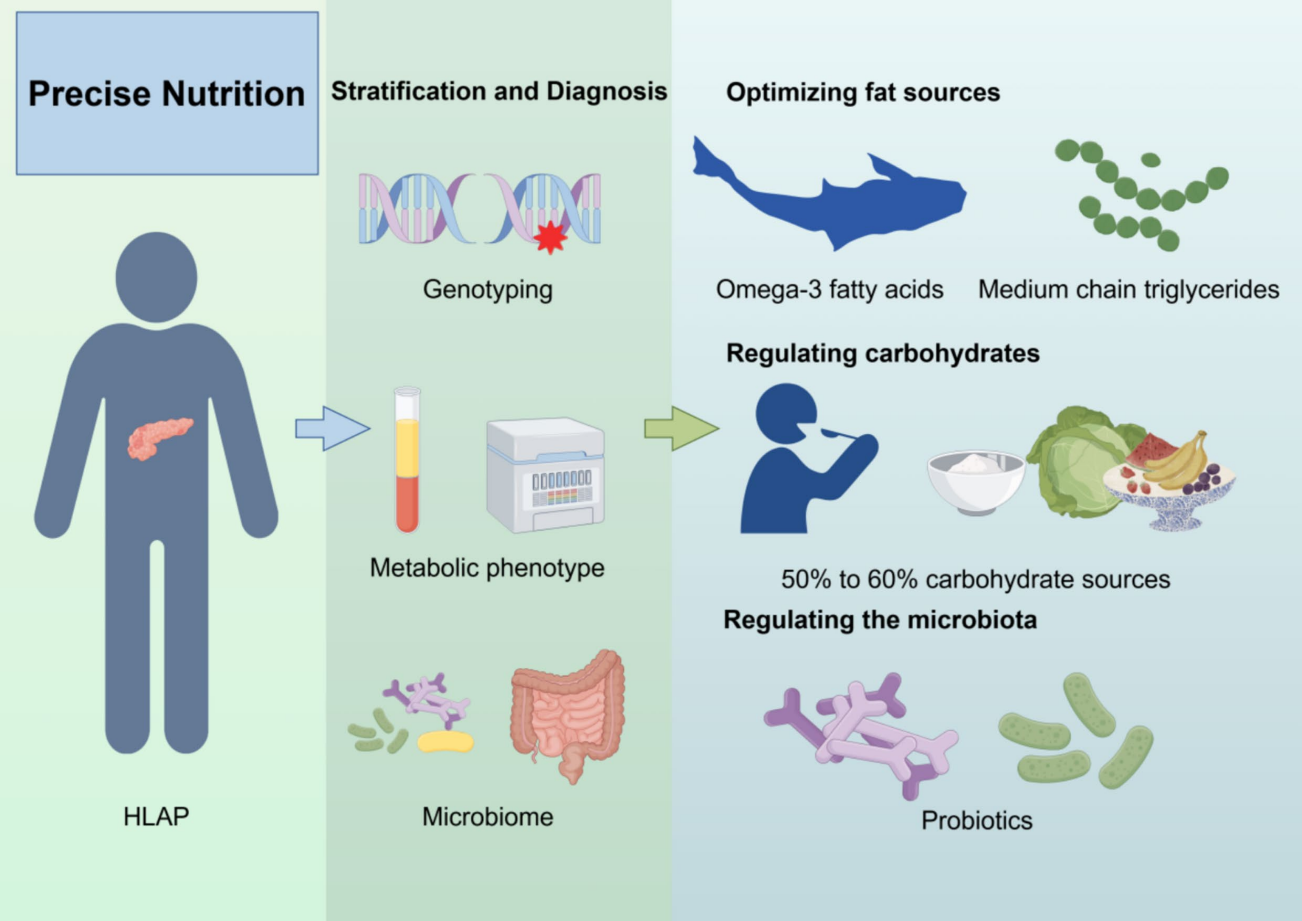
Precision nutrition strategy for HLAP patients. The diagram outlines four components of precision nutrition: (1) lipid source optimization, (2) macronutrient regulation, (3) gut microbiota modulation, and (4) genotype-based dietary adjustment. Created with Figdraw.com.

Precision nutrition emphasizes individualized nutritional interventions based on lipid metabolism characteristics, genetic susceptibility, inflammatory status, and gut microbiota composition to optimize triglyceride levels, reduce pancreatic inflammation, and promote pancreatic recovery (Figure 2) (106).

To facilitate clinical translation and decision-making, a personalized nutrition framework for HLAP management can be constructed with four key components: (1) lipid source optimization, (2) macronutrient regulation, (3) gut microbiota modulation, and (4) genotype-based dietary customization. Each component is tailored to the patient’s metabolic and genomic profile.

First, optimizing fat sources is crucial. Saturated and trans fats should be avoided, while foods rich in omega-3 fatty acids (e.g., deep-sea fish, flaxseeds, walnuts) should be increased (107, 108). Omega-3 fatty acids reduce inflammation and improve lipid metabolism (109). Additionally, medium-chain triglycerides (MCTs), which are directly metabolized by the liver and do not require pancreatic lipase, may serve as a safer fat source for HLAP patients (110). Second, carbohydrate intake should be regulated, with total carbohydrate intake kept below 50–60% of daily calories and primarily sourced from whole grains, fruits, and vegetables rich in fiber (111). One study suggested that replacing 1% of energy intake from carbohydrates with fat sources could reduce serum TG levels by an estimated 1–2% (112).

Regulating gut microbiota is another key component of precision nutrition. Gut dysbiosis in HLAP exacerbates inflammation, so supplementing with probiotics (e.g., Bifidobacterium, Lactobacillus) and prebiotics (e.g., fructooligosaccharides, inulin), as well as consuming foods rich in SCFA (e.g., oats, nuts), may help maintain gut barrier function and reduce enterogenic infections (113). Furthermore, personalized nutrition interventions should incorporate genomic and metabolomic profiles. For instance, patients with APOA5 gene mutations should strictly limit saturated fat intake and increase omega-3 fatty acid consumption (48). Meanwhile, in individuals with PPARG gene mutations, omega-3 fatty acids have been shown to regulate lipid metabolism, significantly reducing LDL-C, total cholesterol, and serum TG levels within 3 months (Table 3) (114). This integrative framework allows clinicians to match specific nutritional components with the patient’s phenotype and genotype, forming the basis for truly personalized dietary therapy. Overall, precision nutrition offers an individualized strategy for HLAP management. By integrating lipid metabolism regulation, inflammation control, gut microbiome balance, and genetic analysis, tailored nutritional plans can be developed to minimize pancreatic damage, enhance treatment efficacy, and improve long-term health outcomes while reducing HLAP recurrence.

**Table 3:** Precision nutrition strategy framework.

Strategy	Interventions	Mechanism	Recommendation basis
Fat source optimization	Increase omega-3 (deep-sea fish, flaxseeds); replace long-chain fats with MCTs	Reduces inflammation; bypasses pancreatic lipase (107–110)	Safe source of fat
Carbohydrate regulation	Total carbs ≤50–60% of calories; high-fiber sources (whole grains, vegetables)	Reduces postprandial TG spikes (111, 112)	Replacing 1% carbs with fats lowers TG by 1–2%
Gut microbiota modulation	Probiotics (Bifidobacterium/Lactobacillus), prebiotics (FOS), SCFA-rich foods (oats, nuts)	Reduces bacterial translocation (113)	Critical for severe HLAP with gut dysfunction
Genomic integration	APOA5 mutations: restrict saturated fats; PPARG mutations: omega-3 supplementation	Corrects lipid metabolism defects (48, 114)	Requires dynamic metabolomic monitoring

Despite the promising theoretical and experimental foundation, the clinical implementation of precision nutrition in HLAP remains at an early stage. Translating these strategies into routine practice requires a critical evaluation of existing barriers.

## 5 Limitations and future directions

First, the heterogeneity of HLAP pathophysiology (e.g., genetic background, metabolic phenotypes, and secondary factors) results in insufficient evidence for individualized interventions. Most existing studies focus on “triglyceride thresholds,” with limited stratified research on lipid tolerance dynamics, specific fatty acid effects, and varying nutritional needs across different disease phases (acute vs. chronic). Second, technical barriers exist in integrating multimodal data, such as real-time metabolic monitoring (lipidomics, gut microbiota metabolites), clinical adoption challenges, and the absence of quantitative models for gene–environment interactions, restricting dynamic precision adjustments. Additionally, clinical implementation faces challenges such as balancing strict fat restriction in the acute phase with the risk of malnutrition, poor dietary adherence in the chronic phase, and the lack of multidisciplinary collaboration mechanisms. Moreover, several practical barriers hinder the clinical translation of precision nutrition. High costs and limited accessibility of genomic and metabolomic testing restrict large-scale screening, especially in resource-limited settings. Real-time monitoring of lipid metabolism and gut microbiota remains technically complex and is not yet feasible in most clinical workflows. To address these gaps, future studies should incorporate stratified randomized trials based on genetic and metabolic profiles, develop point-of-care tools for dynamic lipid and microbiota monitoring, and apply predictive models (e.g., machine learning) to guide individualized nutrition. Real-world studies assessing feasibility, adherence, and cost-effectiveness will be essential to support clinical translation.

## 6 Conclusion

HLAP is a complex disease requiring multidisciplinary collaboration. Precision nutrition, an emerging therapeutic strategy, integrates genomics, metabolomics, and gut microbiome data to provide personalized nutritional interventions. With advances in technology and further clinical research, precision nutrition has the potential to significantly improve HLAP treatment outcomes, enhance patient prognosis, and reduce recurrence rates.
